# Long term evaluation of a multidisciplinary trigeminal neuralgia service

**DOI:** 10.1186/s10194-022-01489-7

**Published:** 2022-09-03

**Authors:** Sanjeet Singhota, Nana Tchantchaleishvili, Jianhua Wu, Ludvic Zrinzo, Lewis Thorne, Harith Akram, Joanna M. Zakrzewska

**Affiliations:** 1 Medical School, University of Birmingham, Birmingham, B15 2GW UK; 2 Current address: The Royal London Hospital, London, E1 1FR UK; 3 Facial Pain Department, Royal National ENT & Eastman Dental Hospitals, London, WC1E 6DG UK; 4 Department of Neurosurgery, The National Hospital for Neurology and Neurosurgery, Victor Horsley, University College London Hospitals NHS Foundation Trust, Queen Square, London, WC1N 3BG UK; 5 Bordeaux Neurocampus, University of Bordeaux, Bordeaux, France; 6 School of Dentistry, University of Leeds, Leeds, LS2 9LU UK; 7 Pain Management Centre, The National Hospital for Neurology and Neurosurgery, London, W1T 3BF UK

**Keywords:** Multi-disciplinary management, Neurosurgery, Trigeminal neuralgia, Medications, Care-pathways, Personalised medicine

## Abstract

**Background:**

Trigeminal neuralgia is an episodic severe neuralgic pain and can be managed both medically and surgically. If possible, this should be directed by a Multidisciplinary Team (MDT) of specialised surgeons, physicians, dentists, psychologists and specialist nurses with access to all treatment modalities, which enables patients to make an informed decision about their future management.

**Objective:**

The aim of this study was to review the outcomes of patients managed by an MDT clinic, in a single institute over an eleven-year period.

**Methods:**

A prospective database was used to identify patients with trigeminal neuralgia or its variants who had attended a joint MDT clinic. The electronic notes were examined for demographics, onset and duration of trigeminal neuralgia, medications history, pain scores and details of surgical procedures if any by two independent assessors.

**Results:**

Three hundred thirty-four patients attended the MDT between 2008–2019. Forty-nine of them had surgery before being referred to the service and were included but analysed as a subgroup. Of the remaining patients, 54% opted to have surgery following the MDT either immediately or at a later date. At the last reported visit 55% of patients who opted to have surgery were pain free and off medications, compared to 15.5% of medically managed patients. Surgical complications were mostly attributable to numbness and in the majority of cases this was temporary. All patients who were not pain free, had complications after surgery or opted to remain on medical therapy were followed up in a facial pain clinic which has access to pain physicians, clinical nurse specialists and a tailored pain management program. Regular patient related outcome measures are collected to evaluate outcomes.

**Conclusion:**

An MDT clinic offers an opportunity for shared decision making with patients deciding on their personal care pathway which is valued by patients. Not all patients opt for surgery, and some continue to attend a multidisciplinary follow up program. Providing a full range of services including psychological support, improves outcomes.

## Background

Trigeminal neuralgia (TN) is an episodic severe neuralgic pain, primarily unilateral, affecting the trigeminal nerve and resulting in significant impact on quality of life [1]. By far, the most common associated radiological feature is neurovascular compression of the trigeminal nerve in the root entry zone in the posterior fossa. Up to 4% of patients with multiple sclerosis (MS) present with TN [2]. A very small percentage of patients with TN are found to have a lesion causing compression of the trigeminal nerve such as a tumour or arteriovenous malformations resulting in what is termed secondary TN. In some patients, no neurovascular compression is identified, and these are termed idiopathic TN [3]. Up to 30% of patients may present with autonomic symptoms. These are not as consistent as in patients with a diagnosis of short unilateral neuralgiform pain with autonomic symptoms, or short unilateral neuralgiform pain with conjunctival redness and tearing [4].

TN can be successfully managed medically. Patients with medically refractory TN or who cannot tolerate medications due to side effects can be managed surgically. Recently updated guidelines support both forms of management [5]. Patients undergoing neurosurgical procedures are more likely to be pain free for longer and off all medications [6]. The treatment patients undergo is often influenced by access to the available range of management options [7].

A long-term follow-up study of a mixed cohort of patients showed that 40% end up having surgery, and the remainder obtain reasonable control of their TN using a range of medical therapies [6].

Not surprisingly, it has been demonstrated that a multi-disciplinary approach to managing patients with TN results in better outcomes [8]. In this approach, patients are first phenotyped and medically managed by specialised headache neurologists and then, if necessary, either referred to a neurosurgical clinic or to a joint clinic run by both specialised neurosurgeons and physicians, with a focus on enabling patients to develop their own personalised care plan [9].

In this study, we aim to present long-term experience from a single centre of managing patients with TN in a joint Multidisciplinary Team (MDT) clinic over eleven years.

## Methods

### Setting

The joint neurosurgical MDT clinic for TN is attended by one or more of three specialised neurosurgeons and a physician consultant who is both medically and dentally qualified. It is held once a month with a maximum of six patients attending with a partner or a carer. Prior to attending, all patients undergo meticulous phenotyping by a physician as well as a battery of assessment including a brain MRI with cranial nerves sequences to visualise the trigeminal nerves. Patients are also requested to complete a set of patient related outcome measures at each visit. The diagnosis and MRI findings are reviewed and confirmed by the MDT. The pertinent surgical options (i.e., microvascular decompression, internal neurolysis, glycerol rhizotomy, radiofrequency thermocoagulation and stereotactic radiosurgery), are offered to patients provided there are no contra-indications e.g., medical morbidity. Patients who wished to have stereotactic radiosurgery were warned that pain relief would not be immediate, and that this was therefore potentially not suitable for those with poor pain control, but more suited to those who had manageable pain, or were pain free and had significant side effects with medication. All patients had access to a pain management program which included clinical psychologists and physiotherapist [10, 11] as well as the doubly qualified facial pain team as per local care pathway [9]. Patients are provided with a written information booklet produced by the Brain and Spine Foundation (https://www.brainandspine.org.uk/our-publications/booklets/face-pain/) [12], and a comprehensive letter including all the surgical options with their pros and cons. The Ottawa Decision Aid [13] is provided and marked with the available options, as well as details of a patient support group TNA UK https://www.tna.org.uk/ [14]. Depending on the outcome from the MDT clinic, follow-up appointments are arranged either with the neurosurgery clinic or the physician led facial pain clinic. Patients with recurrence following surgery are seen again in the MDT clinic.

### Sample participants

A prospective database of all patients attending the MDT clinic is maintained and from this the sample was chosen. Outcomes for this study were assessed by reviewing of the electronic medical records by two independent observer (SS,NT). Patients who attended the MDT clinic over a period of eleven years (2008–2019) were evaluated.

#### Inclusion criteria

All patients who would potentially benefit form a neurosurgical procedure. These included patients with classic TN, idiopathic TN, TN secondary to multiple sclerosis with or without concomitant pain. A small number were later reclassified as having, short unilateral neuralgiform pain with autonomic symptoms, short unilateral neuralgiform pain with conjunctival redness and tearing.as their autonomic symptoms were present consistently. The International Classification of Headache Disorders criteria was applied to making the diagnosis [15]. Patients who had a surgical procedure prior to attendance at the MDT were also included in the sample.

#### Exclusion criteria

Patients with secondary TN due to tumours, cysts, arteriovenous malformations or other types of facial pain e.g., temporomandibular disorders, trigeminal neuropathic pain, and glossopharyngeal neuralgia. Patients with incomplete or inaccessible records and patients who were lost to follow up after opting for surgery were excluded.

#### Process

An MDT clinic database kept prospectively, was used to identify eligible patients before reviewing the electronic records. These included outpatient letters and operating notes. The following information was extracted: demographics, source of referrals, duration of trigeminal neuralgia, pain status and drugs used (when referred, prior to the MDT, at the time of MDT, and at the last visit to the service), and the type and total number of surgical procedures patients underwent. Complications were classified according to Landriel-Ibanez model [16].

Patient reported outcome measures (PROMS) from questionnaires had not been digitised by the time of this study and were therefore not included. Pain status was therefore assessed from the letters which include only a summary of the PROMS but allow for the following grading:

- Pain free: no pain, no drugs
- Pain free on drugs
- Pain mild: 1–3/10 – odd twinge now or in last six months daily or only some days, low dose drugs
- Pain moderate: 4–7/10, regular pain attacks daily but can cope on drug
- Pain severe: 8–10/10– every day, frequent attacks, having to eat soft food, high dose drugs

### Analysis

Descriptive statistics were presented for patient characteristics by the type of medical and surgical management they underwent. Frequency and percentages were used for categorical variables and mean (standard deviation) was used for continuous variables. The difference between groups was investigated by two-sample t-test, or ANOVA for continuous variables and chi-squared test for categorical variables.

This was a service evaluation and so patient consent and ethical approval was not required.

## Results

**Fig. 1:**
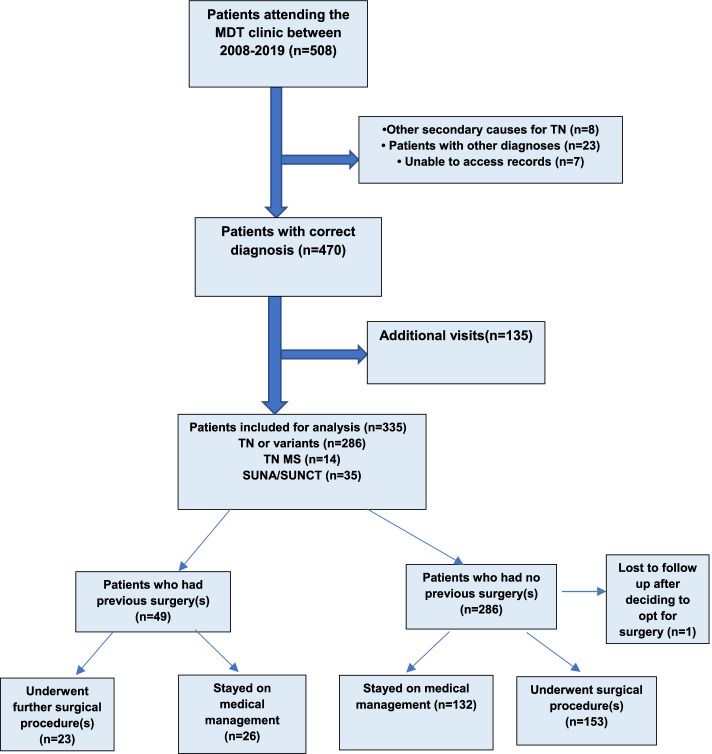
Flow diagram of patient selection. Figure legends: TN = trigeminal neuralgia; GPN = glossopharyngeal neuralgia; SUNA = short unilateral neuralgiform pain with autonomic symptoms; SUNCT = short unilateral neuralgiform pain with conjunctival redness and tearing; MS = Multiple Sclerosis

The diagnosis of those in the study is shown in Fig. 1. A total of 334 patients attended the MDT between 2008–2019, of which 49 patients had surgery prior to attending the MDT clinic (within this institution or externally). These were analysed separately.

The basic demographics are shown in Table 1. On average, patients were seen in the physician led specialist facial pain clinic seven years after onset of symptoms and then 18 months later in the MDT clinic. Although over 60% of patients were referred by primary care practitioners, other referrers included specialists both from the medical and dental specialities. Patients who opted to have surgery where more likely to be on polytherapy, even at the point of referral.

**Table 1:** Basic demographics and status at time of referral

	**Overall**	**Medical treatment only**	**Surgical intervention performed**	**Previous surgical intervention**	***p***
**n**	334	132	153	49	
**TN diagnosis (%)**					0.002
**SUNA/SUNCT**	35 (10.5)	11 (8.3)	12 (7.8)	12 (24.5)	
**TN**	285 (85.3)	118 (89.4)	134 (87.6)	33 (67.3)	
**TN and MS**	14 (4.4)	3 (2.6)	7 (4.6)	4 (8.2)	
**Age at first visit, years, mean (sd)**	58.8 (13.6)	57.8 (12.9)	57.9 (14.1)	64.2 (12.7)	0.011
**Age at MDT, years, mean (sd)**	60.0 (13.8)	58.8 (13.1)	59.1 (14.3)	66.2 (12.5)	0.003
**Male (%)**	119 (35.6)	36 (27.3)	67 (43.8)	16 (32.7)	0.013
**TN duration at first visit, years, mean (sd)**	7.5 (7.7)	6.2 (6.5)	6.9 (7.5)	12.8 (9.6)	< 0.001
**TN duration at MDT, years, mean (sd)**	8.7 (7.9)	7.1 (6.4)	8.2 (7.7)	14.7 (9.9)	< 0.001
**Referrer (%)**^**a**^					< 0.001
**GDP**	62 (18.6)	38 (28.8)	22 (14.4)	2 (4.3)	
**GP**	150 (44.9)	58 (43.9)	71 (46.4)	21 (45.7)	
**Neurologist**	24 (7.2)	5 (3.8)	13 (8.5)	6 (13.0)	
**Neurosurgeon**	21 (6.6)	3 (2.3)	2 (1.3)	16 (34.8)	
**Oral medicine**	33 (9.9)	12 (9.1)	21 (13.7)	0	
**Oral surgery/oral and maxillofacial surgery**	37 (11.1)	15 (11.4)	21 (13.7)	1 (2.2)	
**Pain Medicine**	4 (1.2)	1 (0.8)	3 (2.0)	0	
**Drug at first visit (%)**					< 0.001
**No drugs**	22 (6.6)	4 (3.0)	16 (10.5)	2 (4.1)	
**Monotherapy**	202 (60.5)	102 (77.3)	75 (49.0)	25 (51.0)	
**Polytherapy**	110 (32.9)	26 (19.7)	62 (40.5)	22 (44.9)	

*TN*  Trigeminal neuralgia, *SUNA* Short unilateral neuralgiform pain with autonomic symptoms, *SUNCT* Short unilateral neuralgiform pain with conjunctival redness and tearing, *MS* Multiple sclerosis, *GDP* General dental practitioner, *GP* General practitioner

^a^Missing referrer details for three patients who had previous surgery

Of the group of patients who have not had a surgical procedure prior to the MDT clinic (*n* = 285), a higher proportion opted to have surgery 153 (54%) versus 132 (46%) who opted for medical management only. At the last reported visit of the medically managed patients, only 18 patients (15.5%) were pain free and off drugs with a further 10 patients (8.6%) reporting no pain but on drugs. Whereas in the surgical cohort 84(55%) were pain free and off drugs and a further 20 patients (13%) pain free on drugs.

Table 2 shows that 68% of patients on drug management alone were under improved control with only 11% reporting severe pain compared to 70% at their first visit. Polytherapy had decreased as compared to the first visit, with more on monotherapy with the most common drug being oxcarbazepine.

**Table 2:** Details of medically managed patients

	**1st visit**	**MDT clinic**	**Final follow up**	***p***
**n**	132	132	116	
**Drug (%)**				<0.001
**No drugs**	4 (3.0)	18 (13.6)	25 (21.6)	
**Monotherapy**	102 (77.3)	81 (61.4)	70 (60.3)	
**Polytherapy**	26 (19.7)	33 (25.0)	21 (18.1)	
**CBZ (%)**	54 (40.9)	31 (23.5)	22 (19.0)	<0.001
**OXC (%)**	56 (42.4)	61 (46.2)	49 (42.2)	0.769
**GAB (%)**	8 (6.1)	6 (4.5)	4 (3.4)	0.622
**LAM (%)**	16 (12.1)	32 (24.2)	26 (22.4)	0.050
**PHE (%)**	0 (0)	0 (0)	0 (0)	NA
**BAC (%)**	5 (3.8)	2 (1.5)	2 (1.7)	0.412
**LID (%)**	2 (1.5)	2 (1.5)	5 (4.3)	0.256
**Other drug (%)**	14 (10.6)	15 (11.4)	116 (100.0)	<0.001
**Pain (%)**				<0.001
**Pain free off drugs**	0 (0.0)		18 (15.5)	
**Pain free with drugs**	6 (4.5)		10 (8.6)	
**Mild pain**	14 (10.6)		50 (43.1)	
**Moderate pain**	19 (14.4)		25 (21.6)	
**Severe pain**	93 (70.5)		13 (11.2)	

*CBZ *Carbamazepine, *OXC* Oxcarbazepine, *GAB* Gabapentin, *LAM* Lamotrigine, *PHE* Phenytoin, *BAC* Baclofen, *LID* Lidocaine

The number of patients on drug management alone at the final follow up was lower for various reasons. Four patients were discharged following the MDT, and twelve patients were lost to follow up after the MDT with five of these patients having an open appointment which was not made.

Up to 14% attended another MDT clinic appointment before making their decision. Of the patients who opted for surgery, the most commonly chosen procedure was microvascular decompression (61%) as shown in Table 3. Use of polytherapy prior to surgery was common in all except those patients who had stereotactic radiosurgery. Recurrences occurred after surgical procedures but were lowest in patients who had microvascular decompression (MVD). Of the patients who had an MVD, 12.9% needed repeat surgery with five patients out of twelve opting to have a second MVD. All those who had a recurrence after stereotactic radiosurgery (*n* = 7) had a different procedure. In this group, three patients (43%) had radiofrequency thermocoagulation, two patients (28%) had glycerol rhizotomies and two patients (28%) had an MVD. None of the MS patients had an MVD with 6 (86%) in this group either having a glycerol rhizotomy or radiofrequency thermocoagulation. Two patients had initially failed glycerol rhizotomies due to technical difficulties.

**Table 3:** Results of surgical procedures

	**MVD (%)**	**SRS (%)**	**GLYC (%)**	**RFT (%)**	***p***
**n**	93	15	28	17	
**No of MDT (%)**					0.445
**1**	84 (90.3)	11 (73.3)	22 (78.6)	14 (82.4)	
**2+**	9 (9.7)	4 (26.7)	6 (21.4)	3 (17.6)	
**Drug at first visit (%)**					0.513
Monotherapy	47 (50.5)	9 (60.0)	13 (46.4)	6 (35.3)	
No drugs	9 (9.7)	3 (20.0)	2 (7.1)	2 (11.8)	
Polytherapy	37 (39.8)	3 (20.0)	13 (46.4)	9 (52.9)	
**Length of follow up in years (mean (SD))**	2.53 (2.60)	4.09 (2.51)	4.14 (2.69)	3.16 (2.41)	0.017
**Number reporting any complication**	42 (45)	3 (20)	11 (39)	16 (94)	
**Pain (%)**					<0.001
Pain free off drugs	64 (71.9)	2 (14.3)	10 (38.5)	8 (50.0)	
Pain free on drugs	8 (9.0)	5 (35.7)	7 (26.9)	0 (0.0)	
Mild pain	6 (6.7)	2 (14.3)	5 (19.2)	3 (18.8)	
Moderate pain	6 (6.7)	3 (21.4)	1 (3.8)	1 (6.2)	
Severe pain	5 (5.6)	2 (14.3)	3 (11.5)	4 (25.0)	
**Diagnosis= TN (%)**	82 (88.2)	13 (86.7)	27 (96.4)	17 (100.0)	0.275
**MS (%)**	0 (0.0)	1 (6.7)	3 (10.7)	3 (17.6)	0.003
**MDT to surgery in years (mean (SD)**	0.67 (1.21)	2.03 (2.68)	1.37 (1.76)	1.04 (2.30)	0.014
**Repeat surgery same**	5	0	2	5	
**Repeat surgery other**	7	5	7	0	

*MVD* Microvascular decompression, *SRS* Stereotactic radiosurgery, *GLYC* Glycerol rhizotomy, *RFT* Radiofrequency thermocoagulation, *TN* Trigeminal neuralgia, *MS* Multiple sclerosis

Follow up data was available on 145 of 153 (94.8%) surgical patients. The reasons for lost to follow up was due to: five patients did not have computer records available for the last visit in the service, one patient was lost to follow-up, one patient had recent surgery at the time of data collection, and one patient decided to have the surgery done in another hospital privately. The complications after the differing surgical procedures are shown using the Landriel-Ibanez classification [16] in Table 4. Many of the complications were transient, and some patients had more than one complication. Recurrences are not reported in Table 4.

**Table 4:** Complications after first surgery

Surgical procedure	Number of Complications (%)	Type of deficit
**1**^**st**^**MVD**	**41/93** (44%)	
Grade 1a (no treatment and no drugs)	23t 5p	11t numbness:
		8 mild, 1 moderate, 1 severe,
		1 hemi numbness (lat. Pontine stroke)
		4 p numbness: mild
		4 t hearing: 3 not directly related to surgery
		1 p hearing: haematoma
		2 t blurry vision/diplopia: 1 no ocular pathology, 1 SCA injury
		3 t headache/incision tenderness
		3 t miscellaneous:
		1t back pain after lumbar drain: CSF leak repair
		1t speech/ facial weakness: physio /speech therapy
		1 fall without trauma
Grade 1b (treatment with drugs)	7	5 pulmonary/UTI infections: antibiotics
		1 wound infection: antibiotics
		1 aseptic meningitis/leptomeningitis: steroids
Grade 2a Invasive treatment without GA (Includes local anaesthesia and ± sedation)	12	10 CSF leaks: lumbar drain only 1 Swallowing difficulty: NG tube
		1 bladder catheterisation
Grade 2b invasive under GA	7	2 CSF leaks: mastoid repack
		4 CSF& wound infections: wound revision & lumbar drain
		1 wound infection: cleaned out mastoid area under GA
**1**^**st**^** RFT**	**16/17** (94%)	
Grade 1a (no drugs)	9t 10p	6 t numbness: mild
		8 p numbness: 4 mild, 4 moderate
		1 p reduced sensation
		1t dry eye, earache, pulling sensation in a jaw (in addition to hemi facial numbness)
		1t difficulty opening closing mouth (Osteoarthritis, muscular pain)
		1 t headache
		1 p hearing (not related)
**1**^**st**^** Glycerol**	**13/28** (46%)	
Grade 1a (no drugs)	5t 8p	3 t numbness: mild
		6 p numbness: 2 mild, 3 moderate,1 severe
		1 t swelling (around injection area)
		1 t dry mouth, hyperesthesia
		2 p anaesthesia cornea
**1**^**st**^** SRS**	**3/15** (20%)	
Grade 1a (no drugs)	2p 1t	2 p numbness: 1 mild, 1 moderate
		1 t severe pain

t Transient, p Permanent

*CSF* Cerebrospinal fluid leak, *SCA* Superior cerebellar artery, *UTI* Urinary tract infection, *NG* Nasogastric tube, *MVD* Microvascular decompression, *RFT* Radiofrequency thermocoagulation, *GLYC* Glycerol rhizotomy, *SRS* Stereotactic radiosurgery

Of the 49 patients who had surgery before coming to an MDT 26 patients (53)% did not require further surgery as shown by Table 5. Seventeen patients (35%) who had prior surgery were pain free with only 11(22%) being off medications with most of them achieving this on a single drug. Surgical complications reported in patients who had previous surgery are cumulative and include only long-term complications following their initial surgery(s). Three patients (1%) in this cohort had anaesthesia dolorosa and it was noted in those who had surgery carried out at another hospital. One death was reported in those who had previous surgery. This was a patient who did not respond to previous ablative surgeries with continued severe uncontrolled pain and significant medical co-morbidities. The patient underwent an MVD with no intraoperative complications and was pain free immediately after surgery but died five days later due to a stroke related to his cardiovascular co-morbidities rather then the surgery.

**Table 5:** Outcomes on patients who had prior surgery

	**MVD**	**MVD + ablative**	**Single ablative**	**Multiple ablatives**	***P***
**n**	13	8	15	13	
**Drug at MDT**					
Monotherapy	7 (58.3)	5 (62.5)	6 (42.9)	7 (53.8)	0.799
Polytherapy	5 (41.7)	3 (37.5)	8 (57.1)	6 (46.2)	
**No further surgery**	8 (61.5)	3 (37.5)	9 (60.0)	6 (46.2)	0.639
**Further surgery**	5 (38.5)	5 (62.5)	6 (40.0)	7 (53.8)	
MVD	2 (40.0)	0 (0.0)	3 (50.0)	4 (57.1)	0.327
SRS	1 (20.0)	0 (0.0)	0 ( 0.0)	0 ( 0.0)	
GLYC	1 (20.0)	2 (40.0)	0 ( 0.0)	1 (14.3)	
RFT	1 (20.0)	3 (60.0)	3 (50.0)	2 (28.6)	
**Pain status at last visit**					
Pain free	7 (53.8)	2 (25.0)	3 (21.4)	5 (38.5)	0.341
Mild	2 (15.4)	4 (50.0)	2 (14.3)	4 (30.8)	
Moderate	4 (30.8)	2 (25.0)	8 (57.1)	4 (30.8)	
Severe	0 (0.0)	0 (0.0)	1 (7.1)	0 (0.0)	
**Drug at last visit**					
No drugs	4 (30.8)	2 (25.0)	1 (6.7)	4 (30.8)	0.383
Monotherapy	7 (53.8)	3 (37.5)	7 (46.7)	7 (53.8)	
Polytherapy	2 (15.4)	3 (37.5)	7 (46.7)	2 (15.4)	
**Complications**					
No	9 (69.2)	6 (75.0)	12 (80.0)	8 (61.5)	0.146
Numbness	1 (7.7)	2 (25.0)	0 (0.0)	5 (38.5)	
Anaesthesia dolorosa	1 (7.7)	0 (0.0)	2 (13.3)	0 (0.0)	
Others	2 (15.4)	0 (0.0)	1 (6.7)	0 (0.0)	

Missing pain status for one patient

*MVD* Microvascular decompression, *SRS* Stereotactic radiosurgery, *GLYC* Glycerol rhizotomy, *RFT* Radiofrequency thermocoagulation

## Discussion

In this study, we looked at long-term outcomes of a large cohort of TN patients attending an MDT clinic with both surgeons and physicians being present at the same time with additional structured support of a facial pain team. All data were extracted and analysed by independent assessors (SS, NT and JW).

Fifty four percent of patients with no history of previous procedures opted to undergo surgery, which is in support of the decision analysis study by Spatz et al. [7], where the operation of choice was an MVD. Patients were well informed about the different treatment options. Patients made their own choices based on evidence-based material, which was personalised to their situation and medical co-morbidities. The presence of both a physician and neurosurgeon at the same time, and the availability of all medical and surgical pathways reduces the risk of bias. A satisfaction survey done with the 2018 cohort who attended the MDT clinic shows high satisfaction with this service [17]. As suggested by Slettebo [18], input from the dental specialist is important and all these patients were seen in a postgraduate dental school where a second dental opinion was sought if necessary. The pathway used at this institution is now included in UK National guidelines 2021(https://www.rcseng.ac.uk/dental-faculties/fds/publications-guidelines/clinical-guidelines/) [19].

The pathway used by our team follows the same pathway as described by Heinskou et al. [8] but in their centre, patients are not seen jointly at the neurosurgical clinic a feature that was highly appreciated by patients in this cohort. Overall, better pain control and less drug therapy was needed for patients who underwent surgery, however, those who had surgery elsewhere did not achieve such good results. This is likely to be a result of selection bias since patients who had surgery elsewhere did not attend an MDT and may not have had support from a pain management team. Moreover, these patients were more likely to have had residual symptoms hence the continued follow-up and referral to the MDT clinic.

There is a considerable delay before patients are referred to specialist centres where there is an agreed care pathway with access to an MDT [1]. This pathway is supported by recent updated guidelines on the management of TN [5] and by the Danish Headache Society guidelines [20]. Robust care pathways that include peri-operative protocols, diagnosis, and surgical process result in improved value-based surgery [21]. This approach reduces the chance of low mood and potential for suicide which is up to 2.4% in patients with TN [22], since it provides patients with an individualised care pathway and rapid access to all members of the team should they develop a severe flare up. Although discharged from the MDT clinic patients remain on long term follow up, often by telemedicine, in the facial pain unit which reduces their anxiety, fear and isolation [6, 23].

The complication rate for all surgical procedures appears excessively high. However, the great majority are accounted for by numbness, much of which was temporary, which could be argued is an expected result of trigeminal destructive procedures. Very few patients reported numbness to be severe, long lasting, and intrusive. Three patients reporting hearing problems were not thought to be related to the procedure, but due to prior problems. The rate of CSF leak following MVD is high (17%) but remains in keeping with various reviews reporting an incidence between 0–22% [24]. A change in technique in our institution has reduced the rate of this complication. Otherwise, the surgical results and morbidity rate are in keeping with published series.

There is a need to increase awareness among health care professionals and TN patients of positive outcomes, and that not all patients need surgery to achieve pain control. Nevertheless, patients should be offered the full range of options at an early stage and be referred to specialist centres. The UK NICE guidelines on management of neuropathic pain including TN suggest that patients with TN should be referred to specialist centres once carbamazepine has failed [25]. This at present does not appear to be happening given the long delay for referral. We offer patients the option of a pain management program run by clinical psychologists and physiotherapists to help them live well with TN [10]. All patients have access to an independent clinical nurse specialist with prescribing rights, if they have queries about their medications [11].

Some studies have examined cost effectiveness of neurosurgical procedures, but only one small study comparing medical management versus MVD [25]. Lemos et al. [26] draw attention to the initial higher costs of surgical procedures, but that these reduce over time if no long-term complications occur. Medical therapy does require continued monitoring, as antiepileptic drugs can lead to long term effects including osteoporosis, folic acid deficiency, and drug interactions. Obermuller et al. [27] have showed that post MVD patients are able to return to productive work, and health utilisation is reduced. This may not always be the case with patients on long term medical therapy. In the long term, a registry of all patients using core outcome measures that include the outcome domains of significance to patients, would help guide patients and health care professionals to the optimal care pathway for each individual patient [28].

### Limitations

Some patients were lost to follow up after the MDT. Patients are discharged six weeks after successful surgery, so we cannot be sure that all patients maintained their good outcomes. Although recording of Patient Reported Outcome Measures (PROMs) was carried out, these were not scanned into electronic records, and it was not possible to retrieve all the paper notes. PROMS were very important in making decisions about further management. The Liverpool Adverse Events profile [29] helped the team decide about drug changes and the need for surgery. In this study only pain relief could be measured, and yet quality of life is a crucial outcome and although various scales are available, they are rarely measured [28].

## Conclusion

An MDT clinic with both physician and surgeon in which patients are seen after initial work up provides an opportunity for patients to decide on their personal care pathway which can be altered over time, and which includes support from a multidisciplinary team.

## Data Availability

The data are not publicly available but on reasonable request to the corresponding author, due to their containing information that could compromise the privacy of research participants.

## Abbreviations

MDT: Multidisciplinary Team
MS: Multiple Sclerosis
MVD: Microvascular decompression
PROMs: Patient Reported Outcome Measures

## References

[1] Zakrzewska JM, Wu J, Mon-Williams M (2017). Evaluating the impact of trigeminal neuralgia. Pain.

[2] Zakrzewska JM, Wu J, Brathwaite TS (2018). A systematic review of the management of trigeminal neuralgia in patients with multiple sclerosis. World Neurosurg.

[3] Cruccu G, Finnerup NB, Jensen TS, et al (2016) Trigeminal neuralgia: New classification and diagnostic grading for practice and research. Neurology;87:1-9. 10.1212/WNL.0000000000002840 10.1212/WNL.0000000000002840 PMC4940067 27306631

[4] Maarbjerg S, Gozalov A, Olesen J (2014). Trigeminal neuralgia–a prospective systematic study of clinical characteristics in 158 patients. Headache.

[5] Bendtsen L, Zakrzewska JM, Abbott J (2019). European Academy of Neurology guideline on trigeminal neuralgia. Eur J Neurol.

[6] O'Callaghan L, Floden L, Vinikoor-Imler L (2020). Burden of illness of trigeminal neuralgia among patients managed in a specialist center in England. J Headache Pain.

[7] Spatz AL, Zakrzewska JM, Kay EJ (2007). Decision analysis of medical and surgical treatments for trigeminal neuralgia: how patient evaluations of benefits and risks affect the utility of treatment decisions. Pain.

[8] Heinskou TB, Maarbjerg S, Wolfram F, et al (2019) Favourable prognosis of trigeminal neuralgia when enrolled in a multidisciplinary management program - a two-year prospective real-life study. J Headache Pain (1):23. 10.1186/s10194-019-0973-4 10.1186/s10194-019-0973-4 PMC6734423 30832577

[9] Besi E, Zakrzewska JM (2020). Trigeminal neuralgia and its variants. Management and diagnosis. Oral Surgery.

[10] Daniel HC, Poole JJ, Klein H (2021). Cognitive behavioral therapy for patients with trigeminal neuralgia: a feasibility study. J Oral Facial Pain Headache.

[11] Ghiai A, Mohamed TY, Hussain M (2020). The role of a clinical nurse specialist in managing patients with trigeminal neuralgia. Br J Pain.

[12] Brain and Spine Foundation. Face Pain. https://www.brainandspine.org.uk/our-publications/booklets/face-pain/. Accessed 28 May 2022

[13] O'Connor AM, Drake ER, Fiset V (1999). The Ottawa patient decision aids. Eff Clin Pract.

[14] Trigeminal Neuralgia Association UK. https://www.tna.org.uk/. Accessed 28 May 2022

[15] Anon (2018). Headache Classification Committee of the International Headache Society (IHS) The International Classification of Headache Disorders, 3rd edition. Cephalalgia.

[16] Landriel Ibanez FA, Hem S, Ajler P (2011). A new classification of complications in neurosurgery. World Neurosurg.

[17] Poole J, Mercadante V, Singhota S, Nizam K, Zakrzewska JM (2021). Exploring patient satisfaction of a joint-consultation clinic for trigeminal neuralgia: enabling improved decision-making. Br J Pain.

[18] Slettebo H (2021) Is this really trigeminal neuralgia? Diagnostic re-evaluation of patients referred for neurosurgery. Scand J Pain. 10.1515/sjpain-2021-0045 [published Online First: 2021/08/02] 10.1515/sjpain-2021-0045 34333890

[19] McMillan R, Zakrzewska J, Bahra A, Beecroft E, Besi E, Chong S, Hale A, Durham J, Etherington C, Kitchen N, Lloyd T, Maidment Y, Phillips N, Praveen P, Sharma M, Stamatis L, Tate A, Thorne L. Faculty of Dental Surgery of the Royal College of Surgeons of England. Guidelines for the management of trigeminal neuralgia. https://www.rcseng.ac.uk/dental-faculties/fds/publications-guidelines/clinical-guidelines/2021. Accessed 28 May 2022

[20] Schytz HW, Amin FM, Jensen RH (2021). Reference programme: diagnosis and treatment of headache disorders and facial pain. Danish Headache Society, 3rd edition, 2020. J Headache Pain.

[21] McLaughlin N, Buxey F, Chaw K (2014). Value-based neurosurgery: the example of microvascular decompression surgery. J Neurosurg.

[22] Petrosky E, Harpaz R, Fowler KA (2018). Chronic pain among suicide decedents, 2003 to 2014: findings from the national violent death reporting system. Ann Intern Med.

[23] Venda Nova C, Zakrzewska JM, Ni Riordain, Baker SR (2022) “They could have cut my head off and I wouldn’t have cared” - A qualitative study of patients’ experiences and the impact of trigeminal neuralgia. J Oral and Facial Pain Headache (in press) 10.11607/ofph.3110 PMC12539337 36445910

[24] Khan SA, Laulloo A, Vats A (2020). Microvascular decompression: incidence and prevention of postoperative CSF leakage in a consecutive series of 134 patients. Br J Neurosurg.

[25] NICE. Neuropathic pain - pharmacological management (2013) The pharmacological management of neuropathic pain in adults in non specialist settings guideline 173:1–41

[26] Lemos L, Alegria C, Oliveira J (2011). Pharmacological versus microvascular decompression approaches for the treatment of trigeminal neuralgia: clinical outcomes and direct costs. J Pain Res.

[27] Obermueller K, Shiban E, Obermueller T (2018). Working ability and use of healthcare resources for patients with trigeminal neuralgia treated via microvascular decompression. Acta Neurochir (Wien).

[28] Nova CV, Zakrzewska JM, Baker SR, et al (2020) Treatment outcomes in trigeminal neuralgia-a systematic review of domains, dimensions and measures. World Neurosurg X. 10.1016/j.wnsx.2020.100070 10.1016/j.wnsx.2020.100070 PMC7036566 32123867

[29] Besi E, Boniface DR, Cregg R (2015). Comparison of tolerability and adverse symptoms in oxcarbazepine and carbamazepine in the treatment of trigeminal neuralgia and neuralgiform headaches using the Liverpool Adverse Events Profile (AEP). J Headache Pain.

